# Opioid Addiction: Social Problems Associated and Implications of Both Current and Possible Future Treatments, including Polymeric Therapeutics for Giving Up the Habit of Opioid Consumption

**DOI:** 10.1155/2017/7120815

**Published:** 2017-05-18

**Authors:** M. Cristina Benéitez, M. Esther Gil-Alegre

**Affiliations:** ^1^Department of Pharmacy and Pharmaceutical Technology, Complutense University of Madrid, 28040 Madrid, Spain; ^2^University Institute of Industrial Pharmacy, Complutense University of Madrid, 28040 Madrid, Spain

## Abstract

**Background:**

Detoxification programmes seek to implement the most secure and compassionate ways of withdrawing from opiates so that the inevitable withdrawal symptoms and other complications are minimized. Once detoxification has been achieved, the next stage is to enable the patient to overcome his or her drug addiction by ensuring consumption is permanently and completely abandoned, only after which can the subject be regarded as fully recovered.

**Methods:**

A systematic search on the common databases of relevant papers published until 2016 inclusive.

**Results and Conclusion:**

Our study of the available oral treatments for opioid dependence has revealed that no current treatment can actually claim to be fully effective. These treatments require daily oral administration and, consequently, regular visits to dispensaries, which in most cases results in a lack of patient compliance, which causes fluctuations in drug plasma levels. We then reviewed alternative treatments in the available scientific literature on polymeric sustained release formulations. Research has been done not only on release systems for detoxification but also on release systems for giving up the habit of taking opioids. These efforts have obtained the recent authorization of polymeric systems for use in patients that could help them to reduce their craving for drugs.

## 1. Introduction

Drug addiction is a social problem caused by the continued necessity of taking regular doses of substances in order to feel good or avoid feeling bad. These substances are drugs since their intake effects have serious repercussions on the nervous system which modify the psychic system [1].

Drug abuse causes several acute and chronic toxic consequences, such as consumers becoming dependent. The dependence appears when psychical and physical adaptation to the repeated consumption takes place and, as a consequence, the interruption in the intake would trigger the start of the withdrawal syndrome.

Drug addiction is considered an illness when the patient is unable to control his or her addiction, which affects him or her both physically and psychologically, which in turn has negative social repercussions; therefore a treatment to give up on drugs is required.

An overdose or acute intoxication could take place when the amount of substance introduced in the organism is more than the amount he or she can tolerate. This could lead to a coma or even cause death.

Trying to tackle the problem by reducing drug supply would not put an end to it. To prevent drug abuse from happening, it is essential to give people a proper education so that they will be able to act responsibly when coming across drugs. People should learn to confront their lives and their stressful situations without drugs. Hence education is vital in relation to health.

Drugs have always existed and will continue to exist. Drug abuse has thrived throughout the last decades. It has become an actual social problem due to the increase in drug use in bigger quantities and at younger ages. There are a wide range of drugs which are easy to access [2, 3].

Taking into consideration the latest report published by the European Monitoring Centre for Drugs and Drugs Addiction (EMCDDA), in which the latest facts about drug demand and supply are included, it can be clearly seen that new substances are being introduced and more and more patients are being given opiates as treatment [4, 5].

In the United States, one of the main components of the FDA programme is to educate doctors that prescribe opioid analgesics and other sanitary professionals about the proper way of doing so and how to identify suitable patients for these treatments. Furthermore, they are provided with information about how to educate patients, not only about the proper use of opiates, but also about its correct storage and disposal management [6, 7].

Dependence on opioids has become a social problem. Drug misuse is among the 25 leading causes of risk of mortality worldwide [7, 8]. For instance, in the United States there has been an increase in opiates dependence compared to the previous 10 years. This has led the American Government to allocate a reasonably high amount of money to prevent this situation [9, 10].

According to the United States Centres for Disease Control, opioids were responsible for the majority of deaths in 2010 with over 16,500 deaths (out of roughly 40,000 drug overdose deaths) [7]. In relation to this subject, the FDA published the “Guidance for Industry Assessment of Abuse Potential of Drugs” [11]. This document specifically remarked on the study and development of new pharmaceutical technologies with the aim of reducing drug abuse, “abuse-dissuasive pharmaceutical dosage forms,” being then coined.

Are the polymeric treatments included in such dosage forms?

## 2. Methods

A systematic search was conducted in the most common databases (e.g., PubMed, Medline, web pages of EMA, FDA, AEMPS, Infarmed, Vademecum, Web of Science, Science Database, and Cochrane Database) of relevant papers published until 2016 inclusive using such keywords as “opioid” in combination with “addiction,” “polymeric,” or “treatment.”

All papers included in the manuscript evaluate not only the opiate dependence treatments but also the ones employed for giving up the habit of taking opioids; those papers describing purely genetic factors predisposing to addiction or to the likelihood of abstaining from drug substances were excluded.

## 3. Treatments

When focusing on treatments for opiate dependence, it is observed that treatments usually tend to be more effective when the case of drug abuse is identified in its early stages, although, as usual, detailed treatments oscillate between each person [27].

Firstly, detoxification programmes seek to implement the most secure and compassionate way of withdrawing from opiates in order to minimize the withdrawal symptoms (physical and corporal reactions that take place when a person quits taking a substance to which he/she is addicted). Treatment for patients with opioid use disorder has implicit limitations that reduce its effectiveness and the success of health campaigns. One of the drawbacks appears with the gradual decrease of the treatment with agonist opiates (methadone, buprenorphine, etc.), which causes the appearance of withdrawal symptoms in the patient, which in turn contributes to the increase in the possibilities of a relapse [28].

Medication-assisted treatment (MAT) is a comprehensive approach that combines approved medications (currently, methadone, buprenorphine, or naltrexone) with counselling and other behavioural therapies to treat patients with opioid use disorder [29].

Methadone is a synthetic opiate which blocks the effect of heroin and cancels out the withdrawal syndrome's symptoms. It holds a success record on subjects addicted to heroin.

On the other hand, buprenorphine is a recent inclusion in the list of the different options to treat addiction to heroin and other opiates. Buprenorphine, formulated as sublingual tablets, seems to be the most appropriate medicine for use in a wide variety of treatment conditions when compared to the currently available medicines [28].

Since the mid-nineties, sublingual tablets containing single buprenorphine have been available in order to treat opiate dependence in the European Union. However, drug addicts have abused buprenorphine tablets by dissolving and injecting themselves with the resultant solution.

A buprenorphine/naloxone (Suboxone®) combination is also available formulated as sublingual tablets with the advantage of combining two different active substances, helping to minimize any possible abuse [30]. The joint of buprenorphine/naloxone minimizes this abuse because when naloxone is injected, it can neutralize all the effects caused by opiates, making the patient experience acute withdrawal symptoms.

The pharmaceutical sector has made considerable efforts to develop abuse-resistant and abuse-deterrent opioid formulations which cannot be crushed, merged, dissolved, extracted, or adulterated. [6]. The aim of these opioid formulations is to mitigate the euphoric effects produced by misuse and, simultaneously, make sure they are harmless when properly used.

These treatments require daily oral administration and, consequently, regular visits to dispensaries, which in most cases results in a lack of patient compliance [13].

These limitations of standard methadone/buprenorphine treatment have spurred interest in the development of alternative formulations [14, 31]. Investigation groups are proposing other alternatives as drug delivery systems (using polymers as PLA and PLGA), which are detailed in Table 1. Note, however, some of them may not yet be fully licensed for clinical use.

As shown in Table 1, the main objective of the different investigation groups has been to achieve a long time-release of the drug substance to prevent a lack of patient compliance.

The first efforts were made in 1988 when the drug delivery systems had a release time of less than two days. The efforts continued and release systems with a delivery time of 6 months were soon achieved. The United States authorized in 2016 an implantable formulation of buprenorphine (Probuphine®; Titan Pharmaceuticals, Inc.) intended for the treatment of opioid dependent patients [15].

Probuphine is a polymeric matrix composed of ethylene vinyl acetate and buprenorphine in the form of match-stick size implants (26 mm × 2.5 mm) subdermally implanted which deliver buprenorphine over a period of 6 months.

Thus, a sustained-release implantable formulation of buprenorphine has the potential to be a therapeutically favourable alternative to the current practice of daily or every-other-day administration as it eliminates the need for supervision, thus minimizing fluctuations in plasma concentrations and allowing patients to reduce clinic or pharmacy visits.

### 3.1. Opioid Disorder Use: Giving Up the Habit of Taking Opioids

In order to consider the opiate dependent patient fully recovered, once detoxification is granted, making sure that the patient loses the habit of taking drugs is crucial too.

Antagonist opiates are therapeutic tools for giving up the habit of taking opiates (morphine, heroin) used in patients who have already gone through detoxification programmes, together with other therapeutic measures (e.g., psychological orientation) within an integral rehabilitation programme [32].

Naloxone and naltrexone are pure antagonist active ingredients (opiates receptors with no agonist activity). They block the effects produced by morphine, heroin, and other opiates and do not include any “agonistic” or similar to morphine properties, which are characteristics of other opiate antagonists [33–35]. Treatment with naloxone and naltrexone produces neither physical nor psychological dependence. In addition, tolerance to opiate antagonistic effects has not been observed. Both substances reduce the relapse risk and hold back opiate withdrawal. Furthermore, it is not an aversive treatment and does not cause any reactions after opiate consumption [33–35].

In general, for opioid use disorders, naltrexone is mainly used as tablets. This medicine is an opiate antagonist whose pharmacological period of action lasts longer than naloxone and can be administered orally [33, 34]. This characteristic makes naltrexone the most commonly used opiate antagonist for opioid drug disorder use. Oral naltrexone effectively antagonizes heroin, but its utility is limited by the patient noncompliance.

Sustained-release system preparations may overcome this noncompliance limitation [36]. Recent papers published that oral naltrexone treatment does not present the desirable results, since every time a patient does not adhere to treatment the medicine blood concentration can fluctuate [37–40].

In order to minimize the previously mentioned restrictions, other research groups are proposing alternatives to naltrexone treatments, such as the polymeric systems mentioned in Table 2.

Several alternatives to naltrexone treatments include the use of polymeric systems. Poly(lactide), poly(glycolide), and their copolymers form a major category of biodegradable polymers [41]. As observed in Tables 1 and 2, these types of polymers have received tremendous interest for the development of microparticles formulations [42]. Microparticles were made from poly(lactide), poly(glycolide), and their copolymers, which are biocompatible and biodegradable and have been approved by the FDA for human use [41].

Various techniques to entrap bioactive agents into biodegradable polymers are currently available. These techniques include double emulsion, organic phase separation, supercritical fluid, and spray-drying techniques [43, 44]. Among them, the W/O/W double emulsion technique is the most popular method [45] to entrap water-soluble bioactive agents [46]. Microparticles and nanoparticles offer various important advantages compared to conventional pharmaceutical dosage forms, such as (i) the possibility to accurately control the drug release rates over prolonged periods time [47], (ii) ease of administration (using standard needles), (iii) good biocompatibility [48, 49], and (iv) complete biodegradation (avoiding the removal of empty remnants upon drug exhaustion). They can effectively deliver the drug to a target site and thus increase the therapeutic benefits, while minimizing side effects [50]. This is why the practical importance of this type of advanced drug delivery systems is steadily increasing [51] and they are being extensively used in biomedical applications [50, 52, 53].

For this reason, microencapsulation using biopolymeric materials has given rise to many extraordinary advantages both in pharmaceutical and technological aspects and in the reduction in the lack of treatment compliance [54–56].

One of the main benefits of the encapsulation is that polymeric microparticles/nanoparticles are a good system to delivery opioid antagonists in a control rate so as to get a long therapeutic effect in the patient after a single administration [57, 58].

The naltrexone release in these systems mentioned in Table 2 provides therapeutic blood levels of naltrexone over a period of 28 days when administered through an intramuscular or subcutaneous injection [59]

This effort has led to the development of an intramuscular suspension of polylactide-co-glycolide (PLG) microparticles, which has been authorized by the FDA for opioid dependence in the United States. The intramuscular suspension is administered via injection into the gluteus muscle every 4 weeks [59].

As observed in Table 2, another alternative could be surgical implants consisting of a biodegradable solid polymer inserted or implanted under the skin or fatty tissue with the use of local anesthetic. The wound is then sealed with one to three sutures, with the wound being inspected after about 1 week. The two formulations of surgically implanted naltrexone that have been used in the majority of controlled studies are an Australian type with release periods as long as 7 months [19] and a Russian type with a release period of 2-3 months [24]. These systems reach longer delivery times than suspension system [59].

Not only microparticles and nanoparticles are used as alternatives to naltrexone treatments but also heat-sensitive microgels suspensions have been developed [37]. Microgel suspensions have low viscosity and can be easily administered to the patient by injection. When the samples are heated up to the body temperature, the microgels collapse and aggregate into larger structures. These microgel suspensions could release naltrexone for 5 hours with a high burst effect. It should be pointed out that “burst release” has been a limitation in human trials [21, 60].

On the other hand, as previously mentioned, another opioid antagonist is naloxone. Release systems of naloxone administered by subcutaneous injection to achieve effect during a period of several days could provide quality improvements while losing the habit of taking opiates.

In order to obtain a novel drug delivery system, microparticles of naloxone and poly-*ε*-caprolactone (PCL) have been prepared. Poly-*ε*-caprolactone (PCL) is a biocompatible, biodegradable, semicrystalline FDA-approved aliphatic polyester that degrades slowly and, unlike polylactide (PLA) or polyglycolide (PLG) polymers [61, 62], has been used to prepare microparticles by the oil-in-water (O/W) emulsion-solvent. These microparticles were synthesized as a long-acting delivery system for this opioid antagonist [63–65] since they deliver pharmacologically active naloxone at a constant rate for at least 7 days [66].

Antagonist polymeric systems could be a very useful tool for patient compliance and successful rehabilitation of patients by reducing their craving for drugs [67].

## 4. Conclusions

Dependence on opioids has become a social serious problem (especially due to misuse). The current treatments may be not fully effective since the patient should lose the habit of consumption in order to be considered fully recovered.

Medication-assisted treatment is a comprehensive approach that combines approved medications (methadone, buprenorphine, naltrexone, or naloxone) with counselling and other behavioural therapies to treat patients with opioid use disorder.

Up to now, these treatments usually require daily oral administration and, consequently, regular visits to dispensaries, which in most cases results in a lack of patient compliance. This manuscript shows all the efforts performed by the pharmaceutical sector in order to mitigate this limitation.

Research has been done not only on release systems for detoxification (polymeric systems of methadone or buprenorphine) but also on release systems for giving up the habit of taking opioids (polymeric systems of naltrexone or naloxone). These efforts have obtained the recent authorization by the Health Authorities of polymeric systems for use in patients. This could help them to reduce their craving for drugs with the subsequent positive impact on the social problems associated with drug addiction.

## Figures and Tables

**Table 1:** Examples of alternative drug delivery systems to avoid limitations of standard methadone/buprenorphine treatments.

Publication date	Dug substance	System	Release time	Reference
1988	Methadone	Microspheres of PLA	4 days	[12]
1988	Methadone	Microspheres of PGLA	Less than 2 days	[12]
1988	Methadone	Microspheres of PCL-PLA	2 days	[12]
2004	Methadone	Implant of PLGA	7 days	[13]
2004	Methadone	Implant of PLA	1 month	[13]
2004	Buprenorphine	Depot microcapsules PLA-PLGA polymer	6 weeks	[14]
2009	Buprenorphine	Implant of ethylene vinyl acetate	6 months	[15]
2011	Buprenorphine	In situ forming implants of PLGA	55 days	[16]

**Table 2:** Examples of other alternatives for naltrexone treatments.

Publication Date	Polymer	System	Elaboration method	Release time	Reference
2002	Polyethylene glycol-graft-methyl methacrylate crosslinked	Nanoparticles	Copolymerization	30 days	[17]
2003	Poly(L-lactide)	Matrix devices	Compression-molding of microspheres	75 days	[18]
2003	Poly(L-lactide)	Microspheres	Emulsion solvent evaporation	1 month	[18]
2004	Poly(L-lactide)	Implant	—	1 month	[19]
2006	Polylactide-co-glycolide	Microspheres	O/W emulsion solvent evaporation	1 month	[20]
2006	Poly(L-lactide)	Microspheres	emulsion solvent evaporation	28 days	[21]
2006	Poly(L-lactide)	Matrix devices	Compression of microspheres	360 days	[21]
2009	Bend of poly(N-isopropylacrylamide-acrylamide-vinylpyrrolidone) and PLGA	Nanoparticles	O/W emulsion solvent evaporation	30 days	[22]
2011	Poly (D,L-lactide-co-glycolide)	Microspheres	O/W emulsion solvent evaporation	Up to 25 days	[23]
2012	—	Implant	—	60 days	[24]
2014	Poly [La-(Glc-Leu)] copolymer	Microspheres	O/W emulsion solvent evaporation	28 days	[25]
2014	Poly lactide-co-glycolide	Microspheres	O/W emulsion solvent evaporation	28 days	[26]
